# Development of an In Vitro Blink Model for Ophthalmic Drug Delivery

**DOI:** 10.3390/pharmaceutics13030300

**Published:** 2021-02-25

**Authors:** Chau-Minh Phan, Manish Shukla, Hendrik Walther, Miriam Heynen, David Suh, Lyndon Jones

**Affiliations:** 1 Centre for Ocular Research & Education (CORE), School of Optometry and Vision Science, University of Waterloo, 200 University Avenue West, Waterloo, ON N2L 3G1, Canada; manish.shukla@uwaterloo.ca (M.S.); hendrik.walther@uwaterloo.ca (H.W.); miriam.heynen@uwaterloo.ca (M.H.); david.suh@uwaterloo.ca (D.S.); lwjones@uwaterloo.ca (L.J.); 2 Centre for Eye and Vision Research (CEVR), 17W Hong Kong Science Park, Hong Kong

**Keywords:** contact lenses, drug delivery, eye model, in vitro

## Abstract

Purpose: The purpose of this study was to develop an advanced in vitro blink model that can be used to examine the release of a wide variety of components (for example, topical ophthalmic drugs, comfort-inducing agents) from soft contact lenses. Methods: The model was designed using computer-aided design software and printed using a stereolithography 3D printer. The eyelid and eyeball were synthesized from polyvinyl alcohol and silicone material, respectively. Simulated tear fluid was infused through tubing attached to the eyelid using a syringe pump. With each blink cycle, the eyelid slides and flexes across the eyeball to create an artificial tear film layer. The flow-through fluid was collected using a specialized trough. Two contact lenses, etafilcon A and senofilcon A, were incubated in 2 mL of a water-soluble red dye for 24 h and then placed on the eye model (*n* = 3). The release of the dye was measured over 24 h using a tear flow rate of 5 µL/min. Results: Approximately 25% of the fluid that flowed over the eye model was lost due to evaporation, nonspecific absorption, and residual dead volume. Senofilcon A absorbed more dye (47.6 ± 2.7 µL) than etafilcon A (22.3 ± 2.0 µL). For etafilcon A, the release of the dye followed a burst-plateau profile in the vial but was sustained in the eye model. For senofilcon A, the release of the dye was sustained in both the vial and the eye model, though more dye was released in the vial (*p* < 0.05). Overall, the release of the dye from the contact lenses was higher in the vial compared with the eye model (*p* < 0.05). Conclusion: The blink model developed in this study could be used to measure the release of topical ophthalmic drugs or comfort agents from contact lenses. Simulation of a blink mechanism, an artificial tear film, and nonspecific absorption in an eye model may provide better results than a simple, static vial incubation model.

## 1. Introduction

The development and commercialization of new ophthalmic products require extensive testing for safety and efficacy. While human trials ultimately remain the gold standard, the path to reaching this stage is arduous and extremely expensive. Early-stage in vitro testing can help companies vet promising ideas early in the development cycle, thereby reducing the cost of R&D later on. In addition, in vitro studies can help elucidate underlying properties and mechanisms that contribute to a product’s efficacy [1], which leads to improved product development. The use of in vitro models has also become increasingly attractive during the recent COVID-19 pandemic, where physical distancing has limited access to human studies.

In order to provide useful and predictive data, in vitro eye models should mimic the in vivo conditions as much as possible. The simplest of eye models are glass vials containing a predetermined volume of artificial tear fluid [2,3,4,5,6,7,8,9,10]. Vials are relatively inexpensive, easy to use, and widely accessible. However, this model is too simplistic to mimic the eye, as certain key factors are absent, such as low tear volume, tear flow, intermittent air exposure, and blinking. Consequently, several physiologically relevant eye models have been developed by various groups to provide better in vitro testing platforms [4,9,11,12,13,14,15,16,17,18,19,20,21,22,23,24]. Some studies have shown that results obtained using these advanced eye models are different from those obtained using static vials [12,13,15,16,17,24]. 

Our group, in particular, has focused on the development of an in vitro eye model for evaluating the performance of contact lenses [12,13,15,16,17,24,25]. One of the key advantages of our platform is that a full-sized contact lens can be mounted in a vertical position, as it would be in vivo [12,13,15,16,17,24,25]. The latest iteration of the model simulates air exposure, tear flow, an artificial tear layer, and blinking [24], which are key factors in adequately simulating tear film deposition on lens materials [24]. However, one of the drawbacks of this blink model is that it lacks the ability to effectively capture the flow-through fluid, which is critical in evaluating the release of agents from contact lenses.

Drug delivery using contact lenses has become an increasingly popular concept due to its numerous advantages. Eye drop formulations suffer from extremely low bioavailability due to several ocular barriers such as tear flow, blinking, and nonspecific absorption [26,27]. The use of contact lenses for drug delivery overcomes the aforementioned barriers, resulting in improved efficacy [26,28,29]. In addition, contact lenses can also be designed to provide sustained drug release over several days [26,28,29], which eliminates the need for multiple dosing of eye drops per day or week and may increase patient compliance. Despite the progress in this area, the amount of work in developing advanced in vitro eye models to test drug delivery from lenses remains limited [13,14,15,16,17]. Therefore, the aim of this study was to develop an in vitro blink model that could be used to measure the release of a dye from contact lenses, demonstrating its utility to aid the in vitro examination of a potential ocular drug delivery system.

## 2. Methods

### 2.1. Contact Lenses

Two commercially available daily disposable contact lenses were tested; one conventional hydrogel (etafilcon A (1-Day Acuvue Moist, Johnson & Johnson Vision, Jacksonville, FL, USA)) and one silicone hydrogel (senofilcon A (Acuvue OASYS, Johnson & Johnson Vision)). All lenses had a dioptric power of −3.00 and a base curve of 8.5–8.7 mm, and they were obtained from the manufacturer in the original packaging. They were removed directly from the blister package and rinsed in 1×phosphate-buffered saline (PBS) before use. The properties of the contact lenses are detailed in Table 1.

### 2.2. Blink Model

**Figure 1:**
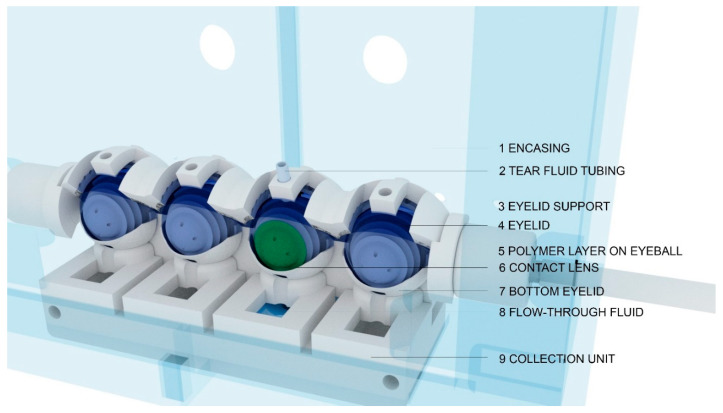
Design and assembly of the blink model. The downward motion of the eyelid spreads a simulated tear solution, which is supplied through the tubing that is attached to the eyelid support, over the eyeball, and a contact lens. The flow-through fluid is then collected in the collection unit located beneath the eyeball.

**Figure 2:**
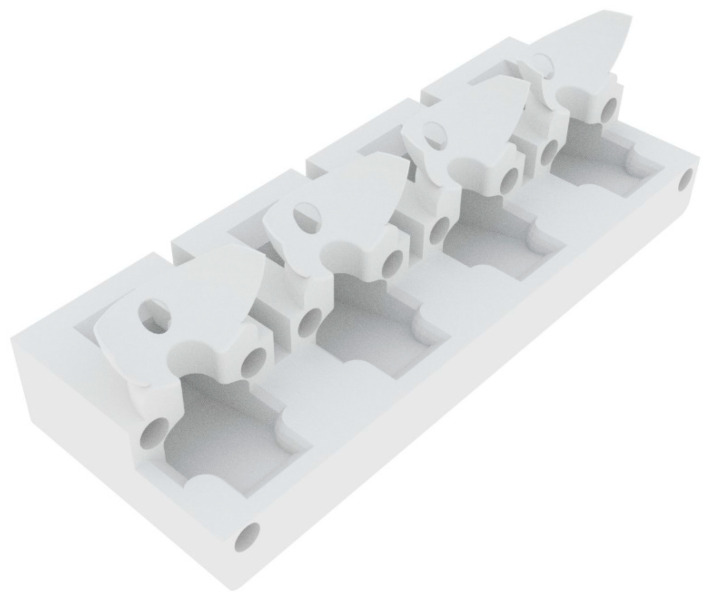
The collection unit allows the fluid to flow from the lower eyelid into the tray located beneath the eyeball.

The fabrication and assembly of the previous iteration of the blink model have been described in a previous study [24] (Video S1, Supplementary Materials). In brief, the eyeball was fabricated using a combination of 3D-printing and molding techniques. For this study, the front surface of the eyeball was coated with a silicone material (Alumilite High Strength 3 Silicone Rubber, Polytek Development Corp., PA, USA) to limit the absorption of the dye. The eyelid was made from a polyvinyl alcohol (PVA) material, which has been described previously [24]. The eyeballs, lower eyelid, and collection unit were printed using a hydrophobic UV-polymerizable resin on an SLA (stereolithography) printer (Photon S, Anycubic, Shenzhen, China) to ensure water-sealed parts. The collection unit was designed to allow the tear film to flow from the eyeball into the wells. The entire system, shown in Figure 1, was housed inside a chamber to maintain a stable humidity during the experiment. A schematic diagram of the collection unit is shown in Figure 2.

### 2.3. Flow and Blink Speed

A commercial syringe pump (PHD ULTRA, Harvard Apparatus, Holliston, MA, USA) was used to infuse the eye model with simulated tear fluid. In this study, phosphate-buffered saline (PBS) was used as the simulated tear fluid. The flow rate was set to 5 µL/min (7.2 mL/24 h), and the blink speed was set to 1 blink/10 s.

### 2.4. Uptake and Release Study

A water-soluble red dye (ClubHouse Red Food Colouring, McCormick & Company, Baltimore, MD, USA) (1.0048 mg/µL) was used as a model agent to visualize the flow-through fluid. The dye consists of a mixture of water, propylene glycol, color (FD&C Red #40), citric acid, and sodium benzoate. The contact lenses were immersed in 2 mL of the dye solution for 24 h at room temperature. After the incubation period, the lenses were placed on the eye model or in a vial containing 2 mL of PBS over 24 h (*n* = 3) at room temperature with gentle shaking. At *t* = 0.5, 1, 2, 4, 8, 12, and 24 h, 20 µL of the sample was withdrawn from the elute and placed into wells of 96-well microplate containing 80 µL of PBS. For the vial study, 20 µL of the sample was withdrawn and replaced by the same amount of fresh PBS to maintain sink conditions. The samples were processed similarly. The absorbance was measured at 520 nm using the SpectraMax M5 UV-Vis Spectrophotometer (Molecular Devices, Sunnyvale, CA, USA). A standard curve of the dye was generated from 0 to 100 mg to determine the amount of dye in the contact lens elute.

### 2.5. Extraction of Dye from Lenses and Eyelid

A set of etafilcon A and senofilcon A lenses were incubated in a 2 mL solution of dye for 24 h. After the incubation, the lenses were extracted with 5 mL of 1:1 acetonitrile/water for 24 h with gentle shaking. Then, 100 µL of this solution was withdrawn, and the absorbance was measured. A set of control lenses without dye was also extracted using the same set of conditions and used as the baseline control. The same procedure was also conducted to measure the amount of dye absorbed into the PVA eyelid on the eye model. After the incubation period, the PVA eyelids were removed from the system, cut into small pieces, and placed into an extraction buffer for 24 h, and then the amount of dye absorbed was determined.

### 2.6. Statistical Analysis

Statistical analysis and graphs were plotted using GraphPad Prism 8 software (GraphPad, La Jolla, CA, USA). All data were reported as mean ± standard deviation (SD) for *n* = 3. A repeated analysis of variance (ANOVA) was used to determine the differences across lens types, incubation conditions, and time points. Post hoc Tukey multiple comparison tests were used when necessary. In all cases, statistical significance was considered significant for a value of *p* < 0.05.

## 3. Results

The eye model was able to collect approximately 4.5 mL after 24 h at an input flow rate of 5 µL/min, which equates to 7.2 mL per 24 h. Approximately 25% of the fluid was lost to nonspecific absorption to the eyelid, evaporation, or residual dead volume on the eye model.

**Figure 3:**
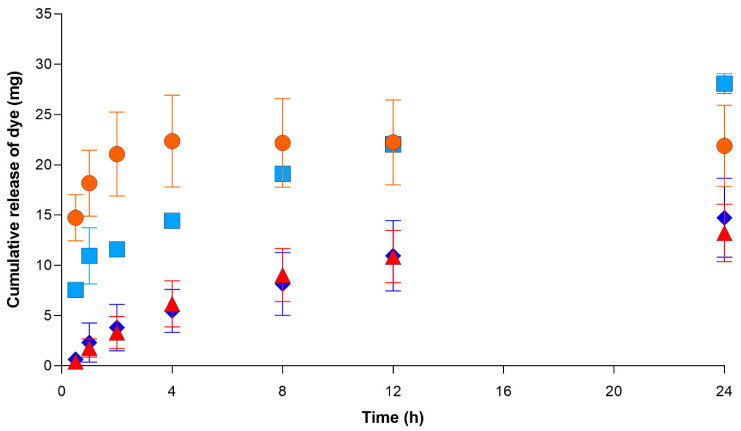
Release of red dye (mg) over 24 h from (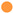) etafilcon A in a vial, (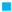) senofilcon A in a vial, (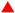) etafilcon A on the eye model, and (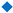) senofilcon A on the eye model (*n* = 3).

The release of the dye (mg) for the vial and the eye model is shown in Figure 3. Overall, there was no statistically significant difference between the cumulative release profiles of senofilcon A and etafilcon A (*p* > 0.05). For the vial, the total amount of dye released after 24 h was 21.8 ± 4.0 mg and 28.0 ± 1.0 mg for etafilcon A and senofilcon A lenses, respectively. In the eye model, the results showed that there was a very slow release of the dye from both lens types. There were no differences in the release kinetics between the two lens types (*p* > 0.05). The total release after 24 h was 13.2 ± 2.9 mg and 14.7 ± 3.9 mg for etafilcon A and senofilcon A lenses, respectively.

**Figure 4:**
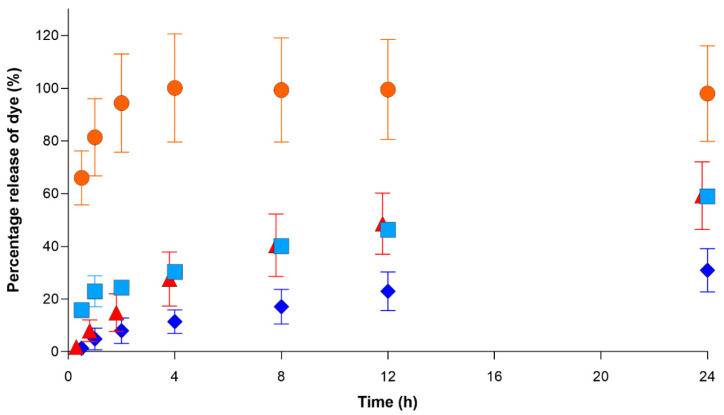
The total percentage release of the dye over 24 h from (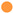) etafilcon A in a vial, (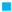) senofilcon A in a vial, (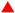) etafilcon A on the eye model, and (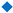) senofilcon A on the eye model (*n* = 3).

The total amount of dye absorbed was 47.8 ± 2.7 mg for etafilcon A and 22.4 ± 2.0 mg for senofilcon A. The percentage release of the dye released from the lenses is shown in Figure 4. In both the vial and the eye model, the total percentage of drugs released for etafilcon A was significantly higher than that for senofilcon A (*p* < 0.05). In the vial, there was an initial burst release of the dye for etafilcon A, which reached 100% of the total release within the first 4 h. In contrast, the release was more gradual for senofilcon A over the 24 h period. The cumulative release and total percentage release of the dye for both lenses were higher in the vial than in the eye model (*p* < 0.05).

**Figure 5:**
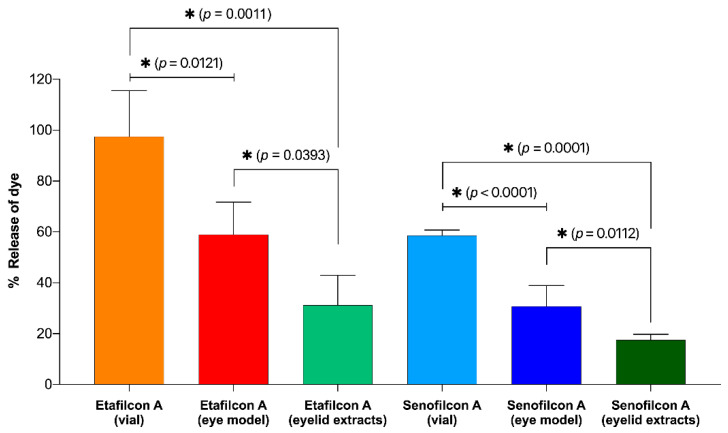
The percentage of dye released from etafilcon A and senofilcon A after 24 h in the vial and the eye model. * Indicates significant differences (*p* < 0.05).

**Figure 6:**
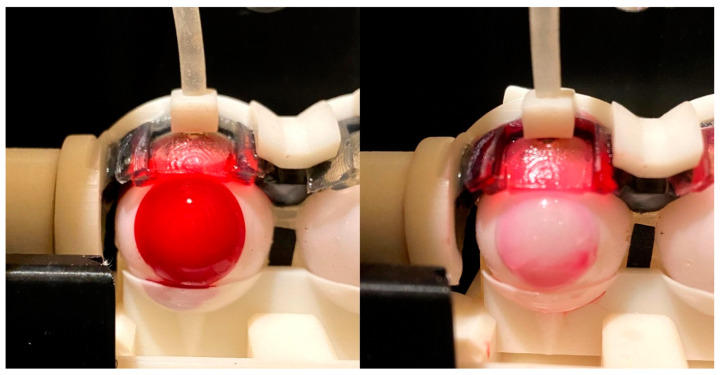
Representative images of etafilcon A at 0 and 24 h on the eye model.

The total percentage of dye released after 24 h is summarized in Figure 5. For the vial study, etafilcon A released 97.5 ± 18.0% of the absorbed dye, whereas senofilcon A released 59.6 ± 2.1% of the total dye absorbed after 24 h. From a visual inspection, the etafilcon A lenses were colorless, whereas there was a light red tint for senofilcon A. In the eye model, etafilcon A released 59.0 ± 12.8% of the total dye absorbed, and senofilcon A released approximately 30.8 ± 8.2% of the dye absorbed. A small portion of the released dye was absorbed into the eyelid, which varied between the two lens types (*p* < 0.05), amounting to 31.2 ± 11.7% for etafilcon A and 17.5 ± 12.8% for senofilcon A. Figure 6 shows a representative picture of the contact lenses at 0 and 24 h on the eye model.

## 4. Discussion

In a previous study, we developed an in vitro model with a physiological blink mechanism [24]. While this model could be used to study drug release, we later discovered several shortcomings that were addressed in the current study. We previously did not consider some of the critical design challenges, such as the intricacies of moving a very small volume of fluid using only gravity and capillary forces, or factors such as dead volume and evaporation. For instance, the lower eyelid component in previous iterations was not able to adequately pool the tear fluid. The buildup of fluid is important as it allows for better control of the fluid movement into a collection unit. If the tear fluid is spread too thinly across a large surface area, then it is prone to evaporation or remains stagnant on the eye model. Additionally, the polylactic acid (PLA) polymers that were used for the 3D-printed parts absorbed a significant amount of fluid, resulting in further fluid loss that could not be collected. The end result was that there were significant inconsistencies in the amounts of fluid that could be collected between various trials.

The focus of the current study was to develop an in vitro blink model that could be used to capture elutes, such as wetting agents or drugs, from a contact lens. The eye model consists of an eyelid that flexes across an eyeball with each blink, which consequently spreads the tear fluid. It is important to note that the tear fluid first pools underneath the eyelid and is only delivered to the eyeball by the blink. Therefore, at a tear flow of 5 µL/min (0.083 µL/s) and 1 blink/10 s, theoretically, there is approximately 0.83 µL of tear fluid that is spread on top of the contact lens with each blink. Similar to previous iterations, the eyelid was fabricated using PVA, a hydrophilic tensile polymer [24]. The eyeball was made from a silicone polymer to ensure that there was no fluid absorption. Furthermore, to avoid fluid absorption into the 3D-printed parts, the model was printed using a water-sealed resin. In addition, compared to previous iterations, many of the components in the current model, such as the eyelids and eyeballs, have also been significantly redesigned and improved for better performance and accuracy [24]. 

This model contained a lower eyelid component to provide structural support to the eyeball, as well as to help hold a contact lens in a stable position. Owing to its thin design (500 µm), the upper eyelid was able to blink over the lower eyelid. The lower eyelid was also designed to allow fluid to accumulate and drip into a special collection tray (see Figure 4) using gravity and capillary forces.

In order to minimize fluid loss due to evaporation, the eye model was encased in an enclosure composed of 3D-printed parts and laser-cut acrylic. The entire system was then placed in a closed humidity-controlled environment during the study (~80% humidity). In theory, the input flow rate at 5 µL/min should amount to 7.2 mL of fluid collected per 24 h. However, the amount of fluid that was collected was approximately 4.5 mL after 24 h, which corresponds to about a 25% loss of fluid. We hypothesize that the loss of fluid in this system was due to evaporation, absorption, and the dead volume of fluid remaining on the eye model.

In regards to evaporation, factors such as temperature, airflow, humidity, composition, and physical properties of the tear fluid can play a role in the loss of fluid [30,31]. For absorption, the tear fluid can be taken up by the eyelid as well as the contact lens. For future studies, if the eyeball is composed of an absorptive material, then a higher amount of fluid loss would also be expected. An important factor that needs to be addressed in future iterations is the dead volume of fluid on the eye model. The current version of the model has a significant amount of contact area, which allows a substantial amount of residual fluid to remain on the 3D-printed parts. As a result, in order to collect enough flow-through fluid required for analysis, we had to select a flow rate that was significantly higher than the physiological flow rate at 1 µL/min (1.440 mL/24 h) [32], in particular for the earlier time points at 0.5 and 1 h.

For a proof-of-concept study with contact lenses, a water-soluble red dye was used as a representative marker. The release profile of the dye from the eye model was compared with a conventional vial incubation. The two contact lenses selected for this study were etafilcon A and senofilcon A. Etafilcon A is a conventional hydrogel with a high water content (58%), whereas senofilcon A is a silicone hydrogel with a much lower water content (38%). Since the marker used in this experiment was a highly soluble dye, we hypothesized that etafilcon A would release the dye much faster than senofilcon A owing to its higher water content.

For the vial-based model system, etafilcon A demonstrated a typical burst and plateau pattern, which has been previously noted in drug release experiments from contact lenses [33,34,35]. In Figure 4, the total percentage of dye released in etafilcon A reached 100% within the first 4 h. In contrast, the release of the dye from senofilcon A was more gradual throughout the 24 h period. The results show that the total percentage of drugs released from etafilcon A is significantly higher than that of senofilcon A in both the vial and eye models (*p* < 0.05), which is in agreement with our initial hypothesis.

Although the contact lenses in the eye model were exposed to more fluid (4.5 mL) than those incubated in the vial (2 mL), the rate of release, as well as the total amount of dye that was released, was significantly lower in the eye model (*p* > 0.05). This difference between the two models could be attributed to several reasons. First, there was a difference in the amount of fluid that the lenses were exposed to during a given time. For the vial, the contact lenses were immediately exposed to 2 mL of solution. As a result, the lenses rapidly expelled the absorbed dye, resulting in a faster and higher release of the dye. In contrast, the contact lenses were exposed to less than 300 µL of solution per hour in the eye model. Consequently, the dye was released more slowly in the eye model, and this is more representative of the situation in an eye wearing a lens. Secondly, in the eye model, there was nonspecific absorption of the dye from the contact lens into the eyelid, which reduced the concentration of dye in the flow-through fluid. Thirdly, a portion of the dye was lost in the residual fluid bound to the 3D-printed parts of the eye model.

Based on the total percentage release after 24 h (Figure 5), for the vial study, etafilcon A released all the dye it absorbed, whereas senofilcon A released only 58.7 ± 2.1% of the dye that was absorbed. In the eye model, both lenses still retained a portion of the total amount of dye absorbed. Etafilcon A released 59.0 ± 12.7% of the absorbed dye, while senofilcon A released approximately 30.8 ± 8.2% of the absorbed dye. These results were validated visually as both lenses still had a slight red tint (Figure 6). Interestingly, the eyelid also absorbed a portion of the red dye that was released. The eyelid absorbed approximately 31.2 ± 11.7% of the dye from etafilcon A and 17.5 ± 12.8% of the dye from senofilcon A. The differences observed in the dye absorption in the eyelid between the two different lens types are likely because the dyes are released more rapidly from etafilcon A. Furthermore, the percentage release of the dye from etafilcon A is also significantly higher than that from senofilcon A. The simulation for nonspecific absorption is an important aspect in understanding the release kinetics of elutes from contact lenses and will be further examined in future studies.

## 5. Conclusions

The current study describes the fabrication of an eye model that could be used to collect flow-through fluid from contact lenses. The amount of dye released from the contact lenses on the eye model was significantly less than that in a vial. For the vial study, the results showed that senofilcon A had a stronger interaction with the dye, as it absorbed more dye and released the dye more slowly over the test period. On the eye model, however, there were no differences in the release of the dye between both lens types. These results suggest that there are differences between the eye model and the vial that warrant further investigation. The absorption of the released dye from the contact lens into the eyelid simulates nonspecific absorption, which may be of significant interest in future research.

## Figures and Tables

**Table 1:** Properties of contact lenses [17].

Categories	1-Day Acuvue Moist	1-Day Acuvue OASYS
USAN	etafilcon A	senofilcon A
Manufacturer	Johnson & Johnson	Johnson & Johnson
Center thickness, mm	0.07	0.085
Water content, %	58	38
Oxygen permeability, ×10^−11^	28	125
FDA group	IV	V (C)
Principal monomers	HEMA, PVP, MA	mPDMS+DMA+HEMA+siloxane macromer +TEGDMA+PVP

HEMA, hydroxyethyl methacrylate; DMA; N,N-dimethylacrylamide; MA, methacrylic acid; mPDMS, monofunctional polydimethylsiloxane; PVP, polyvinylpyrrolidone; TEGDMA, tetraethyleneglycol dimethacrylate.

## Data Availability

The data presented in this study are available in the article or supplementary material.

## Supplementary Materials

The following are available online at https://www.mdpi.com/1999-4923/13/3/300/s1, Video S1: Blink model with a dyed contact lens.
